# An Up-to-Date Literature Review on Ventricular Assist Devices Experience in Pediatric Hearts

**DOI:** 10.3390/life12122001

**Published:** 2022-11-30

**Authors:** Ștefana Maria Moisă, Alexandru Burlacu, Crischentian Brinza, Eliza Cinteză, Lăcrămioara Ionela Butnariu, Elena Țarcă, Alexandru Florinel Oancea, Ioana-Alecsandra Munteanu, Valentin Munteanu, Laura Stătescu, Laura Mihaela Trandafir

**Affiliations:** 1 Department of Pediatrics, Faculty of Medicine, “Gr. T. Popa” University of Medicine and Pharmacy, 700115 Iasi, Romania; 2 Faculty of Medicine, University of Medicine and Pharmacy “Grigore T Popa”, 700115 Iasi, Romania; 3 Institute of Cardiovascular Diseases “Prof. Dr. George I.M. Georgescu”, 700503 Iasi, Romania; 4 Department of Pediatrics, “Carol Davila” University of Medicine and Pharmacy, 020021 Bucharest, Romania; 5 Department of Pediatric Cardiology, “Marie Curie” Emergency Children’s Hospital, 41451 Bucharest, Romania; 6 Department of Medical Genetics, Faculty of Medicine, “Grigore T. Popa” University of Medicine and Pharmacy, 700115 Iasi, Romania; 7 Department of Surgery II—Pediatric Surgery, “Grigore T. Popa” University of Medicine and Pharmacy, 700115 Iasi, Romania; 8 “Saint Spiridon” Emergency Hospital, 700115 Iasi, Romania; 9 Anesthesia and Intensive Care Department, “Grigore T. Popa” University of Medicine and Pharmacy, 700115 Iasi, Romania; 10 Department of Biomedical Sciences, “Grigore T. Popa” University of Medicine and Pharmacy, 700115 Iaşi, Romania

**Keywords:** congenital heart defect, cardiomyopathies, ventricular assist devices

## Abstract

Ventricular assist devices (VAD) have gained popularity in the pediatric population during recent years, as more and more children require a heart transplant due to improved palliation methods, allowing congenital heart defect patients and children with cardiomyopathies to live longer. Eventually, these children may require heart transplantation, and ventricular assist devices provide a bridge to transplantation in these cases. The FDA has so far approved two types of device: pulsatile and continuous flow (non-pulsatile), which can be axial and centrifugal. Potential eligible studies were searched in three databases: Medline, Embase, and ScienceDirect. Our endeavor retrieved 16 eligible studies focusing on five ventricular assist devices in children. We critically reviewed ventricular assist devices approved for pediatric use in terms of implant indication, main adverse effects, and outcomes. The main adverse effects associated with these devices have been noted to be thromboembolism, infection, bleeding, and hemolysis. However, utilizing left VAD early on, before end-organ dysfunction and deterioration of heart function, may give the patient enough time to recuperate before considering a more long-term solution for ventricular support.

## 1. Introduction

Congenital abnormalities, especially congenital heart defects (CHD), are the leading cause of infant mortality associated with congenital disabilities, occurring in almost 1% of live births [1]. This can lead to chronic disability, morbidity, and increased healthcare costs. Significant improvements in cardiovascular diagnosis and cardiothoracic surgery during the past century have boosted the survival of neonates with CHD [2].

The advancement of ventricular assist devices (VADs), envisioned as a temporary bridge to transplant therapy and preferred over the escalation of conventional therapy, has significantly impacted the management of heart failure in CHD and cardiomyopathies [3]. According to the 2019 International Society for Heart and Lung Transplantation (ISHLT) registry report, over one-third of patients receiving transplants are currently being bridged with a VAD [4]. The number of children awaiting transplantation is steadily rising, while the number of pediatric donor hearts remains low [5]. A more significant number of young children with complicated congenital heart problems are now living longer thanks to improved surgical palliation, but, in many cases, the palliated heart eventually fails and requires transplantation. Children placed on the heart transplantation waiting list suffer the most significant risk of mortality pertaining to all those waiting for solid organs [3,4,5].

Pediatric mechanical circulatory support is again receiving attention due to the rising number of sick children with end-stage heart failure [3,4,5]. This literature review will give a general overview of how pediatric VADs are indicated and used currently, and what the future may hold.

## 2. Materials and Methods

The search was performed in Medline (PubMed), Embase, and ScienceDirect databases using several prespecified keywords and MeSH terms (in the case of Medline database): ventricular assist device, pump, artificial ventricles, congenital heart disease, heart defect, cardiomyopathies, children, and pediatric.

The following inclusion criteria were considered for eligibility assessment of individual studies: children aged <18 years were analyzed, full-text available online, and randomized and observational studies reporting original data regarding VAD use in the pediatric population (including efficacy and safety end-points). Studies available only as abstracts, letters, studies that analyzed adult patients, and those which did not meet the inclusion criteria were excluded from the present review. Two independent authors evaluated retrieved studies for eligibility in a two-step process. In the first step, titles and abstracts were screened for inclusion and exclusion criteria. Only studies that fulfilled all inclusion criteria were appraised in the second step as full-text.

When available, we extracted the following data from included studies: year of publication, the number of patients included and their age, the weight of enrolled participants, the type of VAD used, and reported efficacy and safety outcomes.

## 3. Results

Following the screening process and eligibility assessment, included studies were critically discussed and analyzed. Concerning safety outcomes, the main complications associated with VAD use in children encompassed thromboembolism, infection, bleeding, and hemolysis (Table 1). Several representative clinical studies on VAD use in the pediatric population, including reported efficacy and safety end-points, are presented in Table 2.

### 3.1. Berlin Heart Excor

The Berlin Heart EXCOR^®^ pediatric VAD is a mechanical circulatory support device for pediatric patients [9,10,11]. Extracorporeal, pneumatically powered, and available in various sizes, EXCOR is a pulsatile pump. It is implanted outside the chest and attached to the atria, left ventricular (LV) apex, and major vessels. All pumps use the stationary drive system (IKUS) unless driving pressures greater than 250 mmHg are necessary. In November 2019, a brand-new mobile EXCOR Active System for young children was introduced. Only EXCOR is marketed and sold globally for newborns and young children [12].

**Table 2:** Representative clinical trials on VAD use in the pediatric population.

Author, Year	Patients, No.	Age, Mean/Median ± SD	Weight, kg	Type of VAD	Results
Almond, 2013 [9]	204	1.6 (0.5–5.4) years	10.0 (6.5–16.6)	Berlin Heart EXCOR (bridge to heart transplantation)	12 month survival was 75% (including 64% heart transplant recipients) Biventricular device support was associated with early mortality.
Jordan, 2015 [10]	204	18.6 (6.3–64.9) months	10.0 (6.5 to 16.6)	Berlin Heart EXCOR (bridge to heart transplantation)	Adverse neurological events were recorded in 59 patients (28.95%) and were linked to increased mortality (42% vs. 18% in those without neurological events).
Morales, 2017 [13]	204	1.7 (0.3–4.7)—in CHD patients	8.5 (5.2–16.3)—in CHD patients	Berlin Heart EXCOR (bridge to heart transplantation)	CHD patients with VAD had a higher mortality rate than non-CHD patients with VAD (50.9% versus 16.6%, *p* < 0.001).
Fraser, 2012 [14]	48	11.7 (2.6–45.6) months in the VAD cohort	9.2 (3.6–13.6)—in the VAD cohort	Berlin Heart EXCOR (bridge to heart transplantation)	Compared to ECMO patients, those who received VAD had a significantly higher survival rate (10 days vs. 144 days, *p* < 0.001). Key adverse events in the VAD cohort were major bleeding (50%), infection (50%), and stroke (29%).
Reinhartz, 2003 [15]	19	10 (7–14)	31 (17–41)	Thoratec paracorporeal pneumatic VAD	The survival rate was significantly higher in children with cardiomyopathies/myocarditis compared to those with CHD (72% vs. 14%).
Konertz, 1997 [11]	6	5–8 years	3.1–20.5	MEDOS HIA (bridge to transplantation and recovery from cardiac surgery)	Two children died, two children were weaned from VAD, and two children underwent heart transplantation

CHD = congenital heart disease; ECMO = extracorporeal membrane oxygenation; VAD = ventricular assist device.

A study conducted between August 2015 and July 2016 in Suita, Japan, showed satisfactory results on four pediatric patients with idiopathic dilated cardiomyopathy using this device. The EXCOR device was used to implant left ventricular assist devices (LVADs) in patients with end-stage heart failure. At the time of surgery, the typical patient was eight months old and weighed 4.8 kg. All patients made it through the median follow-up of 7.3 months (range, 5.0–10.3). Two patients received heart transplants, while the other two are stable hemodynamic candidates on a waiting list [16].

Case one was of an 11-month-old infant with idiopathic dilated cardiomyopathy (DCM) on mechanical respiratory support and treated with inotropic agents. Her hemodynamic state deteriorated while waiting for the device to be implanted, necessitating immediate central extracorporeal membrane oxygenation (ECMO) with the Endumo^®^ 2000 system (Heiwa Bussan, Tokyo, Japan). Six days later, she underwent EXCOR implantation. Nonetheless, five days after the device implantation, she developed intracranial bleeding that required emergently removal of the hematoma. She was on LVAD support for 310 days without any issues; she received a heart transplant. Paresthesia is still in the left side of the upper and lower extremities, but no central neurological sequelae are present. The second case was of a 4-month-old infant with idiopathic DCM. Her left ventricular ejection percentage (LVEF) was 17% at the time, and her left ventricular diastolic diameter (LVDd) was 50.4 mm. She had cardiac resynchronization therapy (CRT) for left ventricular asynchrony when she was five months old [17]. After the implantation, she received carvedilol and enalapril maleate and remained at home for three years [18]. Despite that, her cardiac function gradually deteriorated. Consequently, at 39 months of age, the EXCOR was implanted. She was on LVAD support for 176 days, then underwent heart transplantation successfully. On day 52 following the transplant, she was released home with no evidence of rejection.

The third case was a 4-month-old infant (weight 4.9 kg) with idiopathic DCM. He was administered inotropic medication (dopamine 3 μg/kg/min, dobutamine 3 μg/kg/min, and olprinone 0.3 μg/kg/min) and was on mechanical ventilator support. A total of 44 days after admission, his ventricular function gradually severed, leading to EXCOR implantation. He was afterward diagnosed with associated Barth syndrome. Cyclic neutropenia is a significant comorbidity of this disease, raising the risk of nosocomial infections [19,20]. The patient needed surgical debridement of the diseased necrotic tissue and negative pressure wound care due to persistent skin infections surrounding the cannula exit sites. He was on the waiting list for heart transplantation while remaining on continuous antibiotic therapy to control the infection.

A 1-month-old baby (weighing 3.0 kg) with idiopathic DCM and cardiogenic shock was the fourth patient involved in the research. This patient experienced severe respiratory failure caused by pneumonia and cardiogenic lung edema. The Endumo^®^ 2000 system started ECMO support immediately, and her left atrium was ventilated via a percutaneous transfemoral catheter through a patent foramen ovale (PFO). Even though these surgeries helped her hemodynamic condition, thoracic compartment syndrome in some way impeded her ability to recover her breathing. As a result, 12 days following the start of ECMO, it was necessary to switch to central ECMO using the Endumo 2000 system and EXCOR pediatric inflow and outflow cannula. The PFO was sealed off, and the atrial venting catheter was withdrawn. Her respiratory function was restored after four days of central ECMO support, enabling conversion to the EXCOR pump under local anesthetic without mechanical right heart support. She is currently in a stable condition without any neurological issues while waiting for a heart transplant.

Another study examining the outcomes of 204 Berlin EXCOR VAD implantations performed in North America between 2007 and 2010 found that some children receiving sustained BiVAD support may have fared better with LVADs alone. The EXCOR Pediatric device assisted 204 patients during the trial [7]. A total of 66 patients (37%) had BiVADs, while 128 (63%) received LVADs. Except for the patients with BiVADs having a more significant percentage of INTERMACS profile one at implantation, both groups had identical preimplantation and implantation characteristics. Additionally, patients with BiVADs had a higher likelihood of being white and having elevated bilirubin levels (>1.2 mg/dL). The two groups’ overall survival rates were noticeably different, with LVADs having a 100 day survival rate of 80% and BiVADs having a 60% survival rate. Compared to patients using LVADs (23%), those on BiVAD assistance had a higher death rate while using the device (32%).

In order to assess the changes in heart transplant waiting list mortality following the adoption of the Berlin Heart EXCOR, a retrospective, single-center analysis was carried out in the Netherlands involving all juvenile patients (18 years) awaiting transplantation. Based on the availability of the BH EXCOR in the center, patients were divided into two groups: era I (1998–2006; not available) and era II (2007 to July 31, 2018; available) [21].

A total of 87 patients, 15 in the era I and 72 in era II, were included. In period I and period II, one patient (7%) and 13 (18%), respectively, needed extracorporeal membrane oxygenator support. Overall mortality (7/15 in era I vs. 16/72 in era II; 47% vs. 22%, *p* = 0.06) and transplantation rates (8/15 in era I vs. 47/72 in era II; 53% vs. 65%, *p* = 0.39) did not differ significantly from one period to the other. Cerebrovascular accidents (CVAs), which affected eight (29%) of the juvenile ventricular assist device (VAD) group, were the primary cause of death in 11 (39%) children. In addition, 14 (or 50%) of the young VAD patients made it to transplantation. The most common adverse events in VAD patients were CVA—occurring in 14 cases and happening in most cases (68%) within 30 days of implantation—and bleeding that necessitated a re-thoracotomy in 14, also occurring in all cases within 30 days of implantation.

The study showed that the BH EXCOR’s introduction to the center had increased the survival of young patients with end-stage heart failure. In era I, end-stage heart failure was the leading cause of death; in era II, CVA was the leading cause of death.

### 3.2. Medos HIA

The Medos HIA (Medos Medizintechnik AG, Stolberg, Germany) is a compact, extracorporeal, pneumatically driven system for left, right, or biventricular support.

A study performed at University Hospital Münster, Münster, Germany, described three neonates that were treated from December 1996 to March 1997 [22]. Dilative cardiomyopathy (*n* = 1), restrictive cardiomyopathy brought on by endocardial fibroelastosis (*n* = 1), and Ebstein abnormality (*n* = 1) were the underlying heart conditions. All the children were in the New York Heart Association’s class IV range and needed an inotrope. The goal was to use the Medos device as bridge to transplantation because recovery was no longer possible in any of the cases. All children presented with compromised end-organ dysfunction; they were intubated and mechanically ventilated.

In the early use of the Medos system, the atria were punctured using the Inflow cannulas of the Medos device. An encircling suture was used to insert left-sided cannulas via the interatrial groove and right-sided cannulas into the right atrium. After surgery, protamine was always used in place of heparin. Full heparinization was initiated 4 to 12 h after surgery during the first implant phase (Medos system), and activated clotting time was maintained between 160 and 200 s. The patients did not receive any antiplatelet medication. All patients could be stabilized with univentricular mechanical support; no perioperative complications were recorded.

All three infants with the Medos system required additional observation of bleeding and evacuation of mediastinal blood clots on postoperative days 4, 7, and 21, respectively. In all neonates, severe thromboembolic events were observed. Two children were afflicted by hemiplegia, but they completely recovered; the third child had an obstruction in the right pulmonary artery and was treated with a recombinant tissue plasminogen activator. As a consequence of the research procedures, two neonates developed infectious complications. In one instance, the infection was successfully treated with antibiotics; in the other, the patient died from multiple organ failure. Two of the three patients carried out a successful bridge to transplant procedures.

A study that looked at a group of 16 children—with a range of ages from 0.1 to 55 months and median weights of 3.2 kg (2.5–14 kg) and 0.9 months (median) showed that the Medos system is secure and efficient for supporting circulation in kids [23]. The Medos system was used 22 times for different purposes: (I) extracorporeal life support (ECLS) in post-cardiotomy heart failure (*n* = 11), (II) ECLS in cardiopulmonary resuscitation (CPR) (*n* = 7), and (III) extracorporeal membrane oxygenation (ECMO) in acute respiratory distress syndrome (ARDS) (*n* = 4). Mechanical circulatory support was provided for an average of 4 days (0–18 days), and 12 patients (or 75%) were successfully weaned off it. However, four of these children (or 25%) died following weaning, with a median survival period of 15 days (6–28 days). The overall survival rate was 50%, and none of the eight survivors suffered neurological damage before being sent home. No severe bleeding, thromboembolic issues, or device failure were reported.

### 3.3. Thoratec

The blood pumps Thoratec^®^, CentriMag^®^, and PediMag^®^ (Thoratec Corporation, Pleasanton, CA, USA) offer a choice for doctors caring for young children with ventricular failure. These centrifugal pumps are magnetically levitated, have low prime volumes and negligible hemolysis, and are permitted for 30 days by the Food and Drug Administration. However, these devices have inherent risks because they necessitate concurrent anticoagulation control [24].

These devices have been successfully used in patients with right-sided failing Fontan physiology. Both decreased inferior vena cava pressure, while CentriMag did not increase superior vena cava pressure in a recently published in vitro model [25].

One study investigated the multicenter use of Thoratec devices in children and adolescents and found similar mortality as in adults, with an increased chance of neurologic complications if the left atria were cannulated [15].

Conway et al. reported a single center ten-year experience with the PediMag device. This study included 37 patients suffering from congenital heart diseases, cardiomyopathy, or after transplant. The median weight was 8.9 kg in the study group, and the median duration of device use was 12 days. A total of 45% of patients required ECMO support before PediMag use. Frequent complications included bleeding (24%), neurologic events (18%), and infection (15%). A total of 21% of children were successfully weaned from support, 42% required long-term VAD support, 12% required transplantation, 9% were switched to ECMO, and 5% died [26]. However, the PediMag seemed inferior to RotaFlow in an in vitro simulated neonatal and pediatric ECMO circuits model [27].

### 3.4. HeartMate III

A new, compact centrifugal ventricular assist device is the HeartMate III. Due to its decreased pump thrombosis and stroke rates, it is widely utilized in pediatrics. HeartMate III was successfully used as a bridge to transplantation in an adolescent with failing Fontan circulation, according to research from Children’s Hospital in Minneapolis, Minnesota [28]. The device is usually implanted by median sternotomy in children, while alternative routes, such as lateral sternotomy, are also possible in adults. In children, the outflow graft is usually connected to the ascending aorta, while in adults, it can also be connected to the descending aorta [29].

O’Connor et al. reported a single-center 3 year experience with this device in adult and pediatric patients suffering from dilated cardiomyopathy or congenital heart defects, some with Fontan physiology. No stroke or pump thrombosis was reported in this study, which the older participants could partly explain. This study’s survival rate was also very high—97% [30].

Encouraging results with this device were also reported by Trezzi et al. [31]. This study included five pediatric patients between January 2016 and September 2020 suffering from idiopathic dilated cardiomyopathy or myocardial failure due to myocarditis. Follow-up was performed for a medium of 1.16 years, and no deaths were reported. No thrombosis or bleeding was reported, either, by using a combined bivalirudin-warfarin approach.

### 3.5. Impella

The Impella device is a catheter-based, miniature ventricular assist device. It is placed by retrograde femoral artery access into the left ventricle across the aortic valve. At a maximum rate of 2.5 to 5.0 L/min, the device helps to maintain systemic circulation by pumping blood from the left ventricle into the ascending aorta. This raises overall systemic cardiac output while virtually immediately and sustainably unloading the left ventricle. Although this device does not have enough pediatric-related expertise, it can help with RV function and is only utilized for short-term ventricular support [32,33].

In one multicentric trial, nearly 160 pediatric patients with acute ventricular failure or dysfunction or those undergoing high-risk catheter-based procedures had their systemic circulation supported by an Impella device [8]. The cases chosen for the implant included ventricular dysfunction with acute CGS in 28 patients, intended support during high-risk arrhythmia-ablation procedures in 4, support of chronic heart failure unresponsive to alternative therapy in 6, and arrhythmia in 1.

Regardless of size or age, 85% of the patients were maintained with Impella 2.5 and CP devices, usually via femoral access. In 38% of cases, repositioning of the device was required. A patient received support for a maximum of 51 days, with a median of 45 h. Most patients had their devices removed because of ventricular recovery (41%) or a change in mechanical support (31%).

Within 30 days after the implant, 12 of the 38 patients (32%) passed away. Six of the twelve deaths happened after the Impella was removed, including five patients who had switched to other devices (ECMO in three cases, VAD in two, and one patient who passed away after bridging to a heart transplant). Eight patients experienced severe reactions, the most important being bleeding and hemolysis.

The implantation of the Impella device unloaded the LV volume, which reduced the LV wall stress, improved the coronary flow, and reduced the myocardial oxygen consumption. Almost 40% of patients were able to be explanted for ventricular recovery, suggesting that using temporary support early on, such as Impella, before CGS and end-organ dysfunction worsen, may give patients enough time to recover before switching to a more permanent form of ventricular support

## 4. Discussion

A ventricular assist device helps to circulate a patient’s blood when the heart can no longer provide hemodynamic support. It is a pump attached between the heart and the aorta or pulmonary artery. This device’s functions include relieving strain on the heart and ensuring adequate peripheral circulation for optimal organ function. This provides the necessary energy for healing processes while reducing heart work and oxygen consumption, allowing the patients to heal the impaired myocardial segments faster or live longer until transplantation [34].

Although the operative techniques have improved, myocardial dysfunction can result from complex congenital heart disease interventions involving the left and right ventricles [35]. This leads to the need for mechanical circulatory assistance in almost 5% of children undergoing open heart surgery [36].

An assessment of the possible hazards and benefits of the intervention should be used to determine the best time to implant a VAD in pediatric patients. The many factors influencing the VAD risk profile, such as patient age/size [37,38,39], anatomy [13,40,41], developmental hemostasis [42], device type [43,44,45], as well as socio-epidemiological factors and conditions related to comorbidities and the severity of the sickness before implantation [13,44,45,46], make this decision-making process even more difficult. Many of these elements interact with one another. It is challenging to determine which of these characteristics causes inferior outcomes because paracorporeal devices are more frequently implanted in younger, smaller patients who are more likely to be sicker, have CHD, and have end-organ dysfunction at the time of VAD installation [44,45,46,47].

Only a few cases of ventricular assist devices in smaller children (20 kg) have been reported. First, when adult-sized devices are used in children, low flow rates may concern thromboembolic problems due to technological considerations [14,37,48,49,50]. Second, right ventricular assist devices need some sort of pulmonary valve, whereas left ventricular assist devices can only be implanted when intracardiac shunts are closed [51].

The demand for pediatric ventricular assist devices is growing despite a lack of sufficiently small valves adapted for children. They provide the opportunity for remedial surgeries for numerous previously incurable kinds of congenital cardiac disease. Moreover, it increases the rate of success of pediatric heart transplantation. A surgical infrastructure that includes a children’s intensive care unit and a constantly open laboratory for monitoring coagulation status is required for optimal outcomes [50,52].

Both pulsatile and non-pulsatile LVADs, sometimes called continuous flow LVADs, are FDA approved.

While continuous flow devices employ a motor to continuously discharge the blood to the systemic circulation at the same rates, pulsatile LVADs mirror the heart’s natural rhythmic movement. Continuous flow LVADs also comprise two categories: axial flow (HeartMate II Left Ventricular Assist Device) and centrifugal flow (HeartWare Ventricular Assist System) [6,53].

These devices have been used most frequently in children with cardiomyopathies, cardiogenic shock, and complex heart malformations, such as the Ebstein anomaly or hypoplastic left heart syndrome.

Although VAD therapy in children was associated consistently with better outcomes in clinical studies [9,11,14], underlying heart conditions could impact outcomes following VAD implantation. Moreover, potential advantages and disadvantages should be considered when selecting a particular type of VAD (Table 3). Concerning this issue, children with CHD had an increased mortality risk compared to non-CHD children (50.9% versus 16.6%) [13]. Therefore, benefits and potential risks should be appraised before indicating VAD therapy in children with specific heart disorders. This is particularly important, as neurological adverse events could be observed in almost 30% of children with VAD, with a subsequent increase in mortality risk [10]. Besides neurological events, major bleeding and infection risk should be accounted for (a 50% incidence in one clinical study) [14]. Consequently, future studies are required to minimize the risk of possible adverse events linked to VAD therapy and to increase the efficacy end-points across all heart disorder subgroups.

## 5. Conclusions

Pediatric patients with end-stage heart failure can successfully transition from early left ventricular assist device support placement to heart transplantation. Additionally, we demonstrated that early LVAD use, prior to the deterioration of heart function and end-organ failure, may provide enough time for recovery before thinking about a more long-term kind of ventricular support. As a result of the development of dependable, more compact LVAD technology with a better morbidity profile may now be used more frequently, leading to better results. This makes VAD support a stand-alone therapy for medically resistant heart failure needing hospitalization, combined with the ability to return patients to their homes.

## Figures and Tables

**Table 1:** Complications associated with Ventricular Assist Device.

Study	Number of Patients	Survival Rate	Principal Adverse Effects	Cause of Heart Failure (Most Frequent)
Adachi et al. [6]	39	95%	Infection	Cardiomyopathy
Zafar et al. [7]	204	80%	Major bleeding, infection, neurologic and renal dysfunction	Congenital heart defects, cardiomyopathy
Rhode et al.	87	61%	Bleeding, neurologic events, infection	Congenital heart defects, cardiomyopathy, after transplant
Dimas et al. [8]	160	68%	Bleeding, hemolysis	Cardiogenic shock

**Table 3:** Synthesis of advantages and disadvantages of ventricular assist devices.

	Type of VAD	Advantages	Disadvantages
1	Berlin Heart Excor	It constitutes a pulsatile pump implanted outside the chest and attached to the atria, left ventricular apex, and major vessels. It could serve as a bridge-to-transplant therapy for children of all ages.	Several disadvantages should be accounted for, including the risk of bleeding (44%), thrombotic (21%), and infectious events (46%), implantation and explantation issues, the need to exteriorize the cannula, and financial problems [9].
2	Medos HIA	It constitutes a compact, extracorporeal, pneumatically driven system for left, right, or biventricular support. Pumps are available in different dimensions and can be used in children regardless of weight and height.	Risk of bleeding events which could require re-intervention and risk of infections (50%) [11].
3	Thoratec	These pumps are magnetically levitated, have low prime volumes and negligible hemolysis, and are permitted for 30 days by the Food and Drug Administration.	Potential adverse events include infectious bleeding, thromboembolic complications (27%), prolonged ventilation, neurologic events, and system malfunction [15]. Also, these devices require anticoagulation control.
4	HeartMate III	It constitutes a compact centrifugal ventricular assist device. It was successfully used as a bridge-to-transplant therapy in adolescents. The device carries a low risk of thrombosis and stroke.	Potential adverse events during follow-up include infectious (11.4%) and bleeding complications (11.4%) and arrhythmias (8.6%) [30].
5	Impella	It constitutes a catheter-based, miniature ventricular assist device, which could be placed by retrograde femoral artery access into the left ventricle across the aortic valve.	The device could be used only for short-term ventricular support. Potential issues should be addressed: vascular access site complications, purge failure, and bleeding events (5.2%) [8].

## Data Availability

Not applicable.
